# *MUSeg‐PSV:* A Real‐Time Deep‐Learning Segmentation of the Prostate Gland and Seminal Vesicles on 29‐MHz Micro‐Ultrasound

**DOI:** 10.1002/bco2.70211

**Published:** 2026-05-20

**Authors:** Ludovica Cella, Marco Paciotti, Giacomo Cavadini, Luca Di Stefano, Pier Paolo Avolio, Vittorio Fasulo, Andrea Piccolini, Cesare Saitta, Edoardo Beatrici, Rafal Kocielnik, Andrew J. Hung, Alberto Saita, Paolo Casale, Massimo Lazzeri, Giovanni Lughezzani, Nicolò Maria Buffi

**Affiliations:** ^1^ Department of Biomedical Sciences Humanitas University Pieve Emanuele Milan Italy; ^2^ Department of Urology IRCCS Humanitas Research Hospital Rozzano, Milan Italy; ^3^ Humanitas University Pieve Emanuele Milan Italy; ^4^ Cedars Sinai Medical Center Los Angeles California USA

**Keywords:** artificial intelligence, deep learning, micro‐ultrasound, MicroUS, real‐time segmentation

## Abstract

**Objectives:**

This study aimed to develop and evaluate MUSeg‐PSV, a real‐time deep‐learning framework for segmentation of the prostate gland and seminal vesicles on 29‐MHz micro‐ultrasound (MicroUS), intended to support anatomical orientation during MicroUS‐guided prostate biopsy and local staging of prostate cancer.

**Patients and methods:**

MicroUS data from 14 patients (21 full‐length videos; 2588 annotated frames) were used to train and test MUSeg‐PSV, a lightweight single‐stage convolutional model based on YOLOv11s‐seg. Two training strategies were compared: a standard configuration with basic augmentations and an augmentation‐rich configuration incorporating advanced spatial and photometric transformations. Model performance was assessed on a held‐out test set using mean average precision (mAP), dice similarity coefficient (DSC), expected calibration error (ECE) and Brier score. Real‐time feasibility was evaluated through inference latency and qualitative assessment of temporal consistency on unseen videos.

**Results:**

The augmentation‐rich model outperformed the standard configuration, yielding a 21% relative increase in mAP and a 4.6‐fold improvement in seminal vesicle AP (0.183 → 0.842). Prostate DSC increased from 0.675 to 0.770 (*p* = 4.4 × 10^−5^), while seminal vesicle DSC remained robust (0.892 vs. 0.876), reflecting a controlled trade‐off with substantially improved SV detection (AP: 0.183 → 0.842). Calibration improved markedly, with ECE decreasing from 0.558 to 0.156 and Brier score from 1.000 to 0.196 (*p* < 0.01). Qualitative evaluation confirmed smooth, temporally coherent overlays and consistent delineation of challenging anatomical regions. End‐to‐end latency (28–35 ms/frame; ~35 fps) demonstrated compatibility with real‐time clinical deployment.

**Conclusion:**

MUSeg‐PSV enables reliable, real‐time multiclass segmentation of high‐frequency MicroUS, providing stable delineation of the prostate and seminal vesicles at clinically viable frame rates. These results support the potential of AI‐assisted MicroUS to enhance operator orientation during MicroUS‐guided prostate biopsy and local staging, and to promote standardised identification of seminal vesicles within prostate cancer diagnostic and staging workflows.

## 1 INTRODUCTION

High‐frequency micro‐ultrasound (MicroUS) at 29 MHz is an emerging imaging modality with increasing relevance in prostate cancer (PCa) diagnostics.^1^ With its near‐histological spatial resolution (~70 μm) and real‐time visualisation capabilities for ‘see‐and‐target’ biopsy guidance, MicroUS provides a potential alternative or complement to multiparametric magnetic resonance imaging (mpMRI), currently regarded as the reference standard for prostate imaging.^1^, ^2^, ^3^, ^4^, ^5^ Unlike mpMRI, which cannot provide live guidance during interventions, MicroUS offers continuous, in‐procedure imaging feedback that directly supports biopsy targeting and therapeutic decision‐making.

Despite its advantages in accessibility, cost‐effectiveness and integration into urologist‐led workflows, MicroUS has yet to achieve widespread clinical adoption.^3^, ^6^ This is largely due to the absence of robust, real‐time, standardised guidance tools for anatomical orientation and target localisation, combined with the steep learning curve associated with interpreting high‐resolution MicroUS images.^1^, ^3^, ^4^ As a result, diagnostic performance remains strongly operator dependent, with substantial interobserver variability.^7^, ^8^ These limitations currently prevent MicroUS from fully realising its potential as a dependable intraprocedural imaging platform and reinforce reliance on mpMRI, which is costly, time‐consuming and does not provide real‐time guidance during interventions.^4^, ^5^

In this context, artificial intelligence and deep learning in particular offers a compelling opportunity to overcome these barriers. Convolutional neural networks (CNNs) have shown high performance in medical image segmentation tasks, including prostate MRI, where they have contributed to improved diagnostic accuracy and consistency.^9^, ^10^, ^11^ Translating these advances to MicroUS in a real‐time setting could provide stable, anatomically meaningful overlays during live scanning, reducing operator dependency, standardising interpretation and directly supporting needle guidance and treatment planning.

Several deep‐learning models have already been proposed for segmentation tasks on MicroUS images, primarily focusing on delineating the prostate gland and, in some cases, the prostatic urethra.^12^, ^13^, ^14^, ^15^ However, there is a significant gap in the automated identification and segmentation of the seminal vesicles. This omission is clinically relevant, as accurate delineation of the seminal vesicles is essential for local staging and for the identification of extracapsular extension into the seminal vesicles, corresponding to T3b stage disease.^16^, ^17^, ^18^, ^19^ Real‐time visualisation of the seminal vesicles on MicroUS could enhance risk stratification, biopsy targeting in the base and periprostatic regions and support intraprocedural assessment of suspected locally advanced disease. Without this anatomical information, the full potential of MicroUS for staging and treatment planning remains underutilised.

To address this unmet need, we developed MUSeg‐PSV (Micro‐ultrasound Segmentation of the Prostate and Seminal Vesicles), a lightweight real‐time segmentation system based on a YOLOv11s‐seg architecture for pixel‐level delineation of both the prostate gland and seminal vesicles on 29 MHz B‐mode MicroUS. This work provides a technical feasibility evaluation of real‐time MicroUS segmentation in a routine clinical setting, while recognising that subsequent larger scale, multicentre studies will be required to establish full clinical generalizability.

## 2 PATIENTS AND METHODS

### 2.1 Study population

MicroUS videos were acquired using the ExactVu™ system (Exact Imaging, Markham, Canada) from patients with suspected PCa, based on elevated prostate‐specific antigen (PSA) levels (>4 ng/mL or rising trend) or abnormal digital rectal examination (DRE), between December 2024 and May 2025.

The final cohort comprised 14 patients contributing 21 full‐length MicroUS videos and 2588 manually annotated frames; four patients had clinically significant cancer confirmed on biopsy. All videos were exported directly from the ultrasound console and irreversibly anonymised in compliance with institutional data governance and privacy regulations.

To reflect real‐world variability, we intentionally retained heterogeneous anatomy and image quality; prostate volume (mL) and presence of a median (third) lobe were recorded when visible to enable subgroup analyses.

Inclusion criteria are as follows: adult males (≥18 years) undergoing MicroUS for clinical suspicion of PCa with complete peripheral‐zone coverage and adequate visualisation, irrespective of gland size or median lobe.

Exclusion criteria are as follows: videos unusable due to severe motion artefact, signal dropout/attenuation or incomplete sweeps and corrupted or incomplete files.

Baseline patient characteristics are reported in Table S1.

### 2.2 Study endpoints

This study aimed to develop and validate MUSeg‐PSV, a real‐time segmentation system for delineating the prostate gland and seminal vesicles on 29 MHz B‐mode MicroUS. The model was designed to achieve diagnostic‐grade accuracy, with dice similarity coefficients (DSCs) comparable to expert manual contours, while maintaining inference latencies on the order of tens of milliseconds per frame. The system was further conceived to integrate seamlessly into live biopsy procedures, overlaying precise anatomical masks without interrupting workflow.

A secondary objective was to evaluate the effect of an augmentation‐rich training configuration compared with a standard baseline on overall model performance. The proposed pipeline was evaluated in terms of accuracy, speed and robustness to image‐quality variations and benchmarked against contemporary segmentation approaches. In addition, calibration metrics were used to assess the reliability of model predictions, ensuring that confidence scores reflect actual segmentation accuracy.

### 2.3 Dataset and image annotation

Frame‐level segmentation was performed using Encord, a collaborative annotation platform supporting dense polygon labelling with full auditability. Two trained urology residents (L.C. and G.C.) created the initial labels, which were subsequently reviewed and corrected by an experienced urologist (G.L.). Inter‐rater agreement between the two annotators, assessed prior to expert adjudication, yielded a Cohen's kappa of 0.84, indicating strong concordance. Two anatomical classes were retained (1) prostate gland and (2) seminal vesicles. A custom Python pipeline converted raw videos into a MUSeg‐PSV‐compatible format. All annotated data were exported in COCO JSON format, along with corresponding JPEG frames and MP4 video files.^20^

To prevent information leakage, the dataset was split at the video level into training (70%), validation (20%) and test (10%) subsets using a fixed random seed, resulting in approximately 1812 training frames, 518 validation frames, and 258 test frames. No frames from the same video appeared in multiple partitions.

### 2.4 Preprocessing and model input preparation

Images were resized to 640 × 640 pixels and pixel intensities were normalised to the [0–1] range. Corresponding segmentation masks were encoded as single‐channel PNGs,^21^ then converted into polygon coordinates as required by the Ultralytics segmentation branch. Class mappings were defined as 0 = background, 1 = prostate and 2 = seminal vesicles. All assets were referenced via a standardised *data.yaml* configuration.

### 2.5 Model architecture and training strategy

MUSeg‐PSV is based on the YOLOv11s‐seg architecture, a lightweight, single‐stage convolutional network optimised for real‐time object detection and segmentation.^22^, ^23^, ^24^ Training was executed on Google Colab using NVIDIA T4 or A100 GPUs with standard deep‐learning libraries (Ultralytics, PyTorch).^24^ The model was trained using stochastic gradient descent over 100 epochs with a batch size of 32 with cosine learning‐rate decay. Early stopping (patience = 15 epochs) monitored validation mean Average Precision (mAP); the best checkpoint was retained.

The following two training regimes were implemented:

- *Standard training*: basic augmentations (horizontal flip, HSV shift, geometric jitter);
- *Augmentation‐rich training*: additional transformations (Mosaic, MixUp, random scaling, rotation, translation, and shear) for improved generalisation.^25^, ^26^

The chosen augmentation suite follows evidence‐based best practices for medical image segmentation. Mosaic and MixUp augmentations have been shown to enhance small‐object recall and reduce overconfidence.^25^, ^26^ Standard geometric and intensity transformations (horizontal flip, rotation, translation, scaling and brightness/contrast adjustments) represent the most empirically supported augmentations for ultrasound and other biomedical modalities.^27^ These transformations simulate realistic probe‐induced variation without distorting anatomical boundaries. More aggressive approaches such as elastic deformations or synthetic image generation were avoided as they may degrade anatomical fidelity.^28^, ^29^

### 2.6 Evaluation metrics and statistical analysis

Model performance was evaluated on the held‐out test set using Intersection‐over‐Union (IoU), DSC and mAP. Class‐wise confusion matrices were computed at the confidence threshold maximising the F1 score. Calibration was assessed using expected calibration error (ECE) and continuous Brier score.^30^

Per‐frame DSC values were calculated via polygonal area overlap between predicted and ground truth masks. Paired per‐frame DSC values from the two configurations were compared using the Wilcoxon signed‐rank test, with 95% bootstrap confidence intervals.^31^

### 2.7 Inference and real‐time deployment

The final outputs of both algorithms, consisting of the video with mask overlays, were also qualitatively reviewed by trained urologists to assess overlay accuracy, smoothness of segmentation and overall comparative performance. The final MUSeg‐PSV models were deployed on full‐length MicroUS videos excluded from training. Real‐time inference was performed at 640 × 640 pixels with a raw model inference time of ≈3.1 ± 0.2 ms per frame on an NVIDIA T4 GPU. Including preprocessing and postprocessing (resizing, normalisation, mask decoding, overlay rendering and video writing), the end‐to‐end pipeline latency was 28–35 ms per frame (~35 fps), suitable for live clinical integration. Segmentation masks were overlaid on grayscale μUS frames and exported as MP4 videos for expert review, with special attention to challenging regions such as the prostate apex and seminal vesicle horns.

All outputs were qualitatively assessed by trained urologists for overlay accuracy, temporal smoothness, and overall segmentation quality.

## 3 RESULTS

**FIGURE 1:**
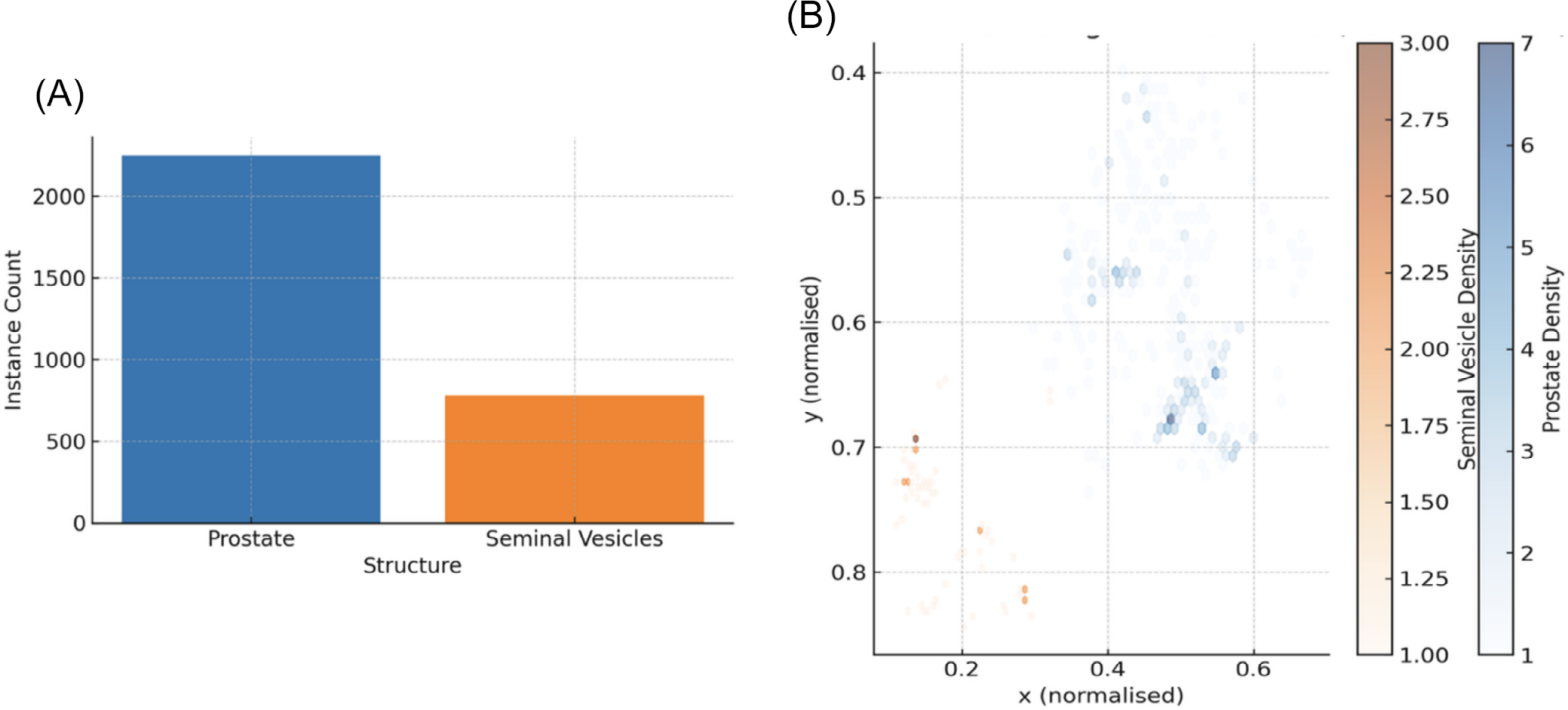
(A) Class frequency distribution of prostate and seminal vesicle annotations in the training set. (B) Spatial dispersion heatmap showing the normalised position of annotated structures across the MicroUS field of view.

The annotated dataset was used to evaluate the performance of MUSeg‐PSV. Overall, the distribution of annotations revealed a moderate class imbalance, with approximately 2250 prostate instances and 780 seminal vesicle instances (Figure 1A). Bounding‐box heatmaps confirmed that the prostate consistently occupied the central field of view, while the seminal vesicles were primarily located in the cranio‐posterior third, occupying a smaller spatial region (Figure 1B). Despite class imbalance and interpatient variability, the dataset supported stable convergence and generalisation. To mitigate under‐representation of minority classes, class‐weighted loss functions were applied to ensure balanced learning across both structures. These measures enabled reliable segmentation of the prostate and seminal vesicles on high‐frequency MicroUS.

### 3.1 Segmentation performance and mAP evaluation

We compared the segmentation performance of the Standard MUSeg‐PSV model with its augmentation‐rich variant. The augmented model achieved a +0.120 increase in mAP 0.5 (+21%) and also improved at more stringent overlap criteria (+0.039 mAP 0.5–0.95), all without added inference latency (Table 1).

**TABLE 1:** Segmentation performance metrics on the held‐out test set for standard and augmented models.

Model	mAPmask (0.50)	mAPmask (0.50–0.95)	Dice/F1peak	Inference speed
Standard	0.575	0.357	0.62	2.9 ms ± 0.2
Augmented	0.695	0.396	0.77	3.1 ms ± 0.2

Abbreviation: MAP, mean average precision.

**FIGURE 2:**
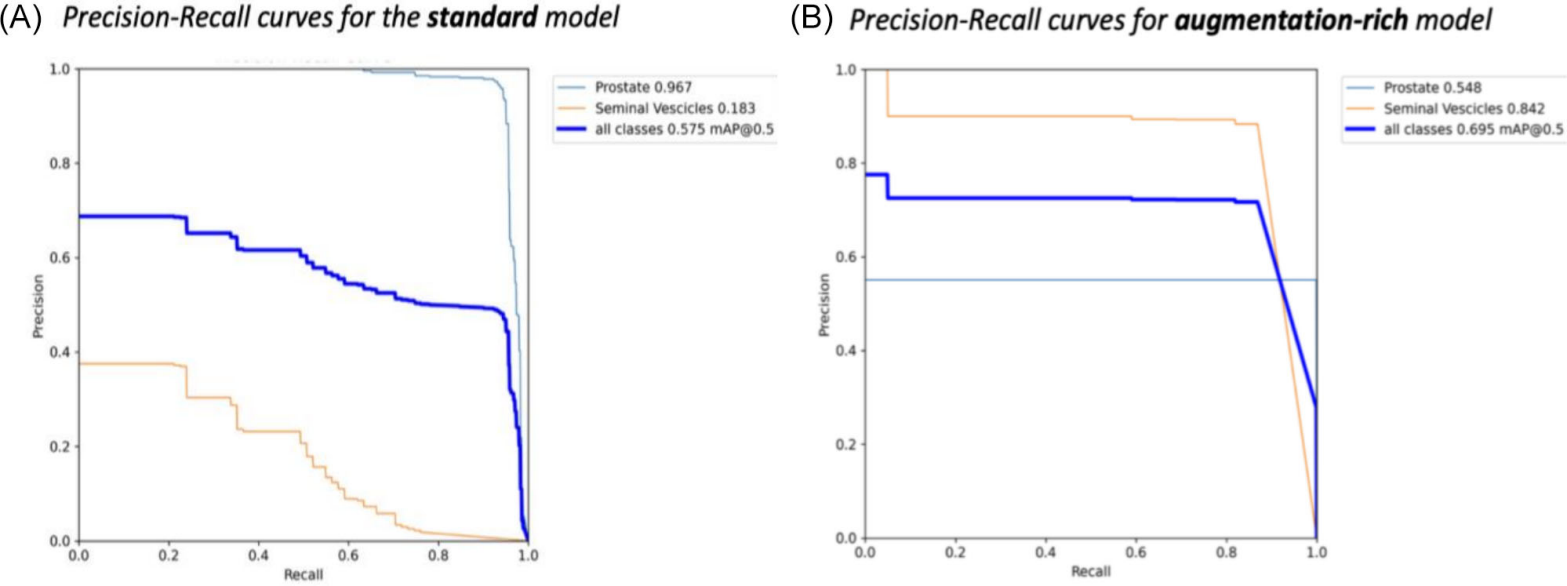
Precision–recall curves for the standard (A) and augmentation‐rich (B) training regimes, illustrating the impact of data augmentation on class‐wise detection performance.

Augmentation led to a 4.6‐fold increase in seminal vesicle AP (from 0.183 to 0.842), while prostate AP decreased from 0.967 to 0.548 (Table 2). Although this represents a trade‐off, overall segmentation quality improved due to the large gains in seminal vesicle delineation (Figure 2).

**TABLE 2:** Class‐wise performance.

Metric	Prostate (AP0.5)	Seminal Vesicles (AP0.5)
Standard	0.967	0.183
Augmented	0.548	0.842

*Note*: AP0.5 = Average precision at an intersection over union threshold of 0.5.

### 3.2 DSC analysis

To further assess per‐class overlap accuracy, we computed the DSC. In the Standard model, seminal vesicle DSC appeared artificially high (0.892) because the model detected fewer seminal vesicles overall but achieved high overlap when it did detect them, whereas prostate DSC was lower (0.675). The augmentation‐rich model achieved more balanced and consistent segmentation, with prostate DSC increasing to 0.770 and seminal vesicle DSC remaining robust at 0.876 (Table 3a). Of note, seminal vesicle DSC decreased marginally from 0.892 (standard) to 0.876 (augmented), a trade‐off discussed in Section 4.

**TABLE 3:** Dice coefficient analysis: (a) class‐wise dice coefficients for standard and augmentation‐rich models; (b) frame‐level dice comparison across the test set.

(a)		
Metric	Standard model	Augmented model
Prostate dice	0.675	0.770
Seminal vesicles dice	0.892	0.876

(b)	
Metric	Value
N frames	69
Dice (AUG) mean ± SD	0.5468 ± 0.4566
Dice (STD) mean ± SD	0.2029 ± 0.4051
ΔDice mean (AUG–STD)	0.3439
95% CI for ΔDice	[0.2146, 0.4721]
Wilcoxon signed‐rank	stat = 270.000, *p* = 4.416e‐05

Abbreviations: AUG, augmented model; CI, confidence interval; SD, standard deviation; STD, standard model.

Additionally, paired per‐frame DSC values across the entire test set confirmed a statistically significant improvement: The augmentation‐rich model achieved a mean DSC 0.55 ± 0.46, compared to 0.20 ± 0.41 for the Standard model, corresponding to an average gain of +0.34 DSC units (95% CI [0.21, 0.47]; Wilcoxon *p* < 0.001) (Table 3b). This frame‐level analysis underscores both higher overlap and greater temporal consistency.

### 3.3 Confidence calibration and model reliability

Prediction reliability was evaluated using confidence calibration curves. ECE decreased markedly from 0.558 (standard) to 0.156 (augmented; *p* < 0.005), indicating that the model's confidence scores became more reliable after augmentation.

### 3.4 Calibration Performance

Calibration performance was further quantified using the continuous Brier score, computed on a 69‐frame random subset (25% of the test set, seed = 42). This subset was used due to the computational cost of extracting per‐pixel probability maps, whereas ECE was calculated on the full test set using aggregated confidence statistics across all predictions.

For the prostate class, the augmentation‐rich model achieved a lower median Brier score (0.049 vs 0.121; W = 742.0; *p* = 0.0054), while for seminal vesicles, the reduction was more pronounced (1.000 → 0.196; W = 11.0; *p* < 0.001) (Table 4).

**TABLE 4:** Calibration performance assessment using the continuous Brier score.

Class	Condition	Mean	Median	95% CI (median)	W	*p*‐value
Prostate	Standard	0.292	0.121	[0.103, 0.475]	742.0	0.0054
Prostate	Augmented	0.375	0.049	[0.030, 0.722]	—	—
Seminal vesicles	Standard	0.884	1.000	[1.000, 1.000]	11.0	<1 × 10^−6^
Seminal vesicles	Augmented	0.511	0.196	[0.146, 1.000]	—	—

**FIGURE 3:**
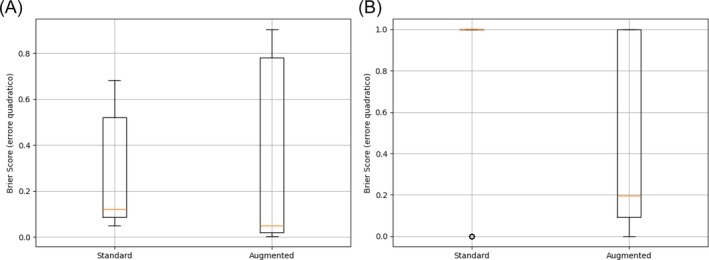
(A) Boxplot of the continuous Brier score for the prostate class. (B) Boxplot of the continuous Brier score for the seminal vesicle class. Lower Brier scores indicate improved probabilistic calibration.

Figure 3A shows reduced median but higher variance for prostate scores, while Figure 3B shows a marked decrease in both median and variance for seminal vesicles. Statistical significance was confirmed via Wilcoxon signed‐rank tests, and 95% bootstrap confidence intervals were used to estimate uncertainty.

### 3.5 Qualitative inference analysis

**FIGURE 4:**
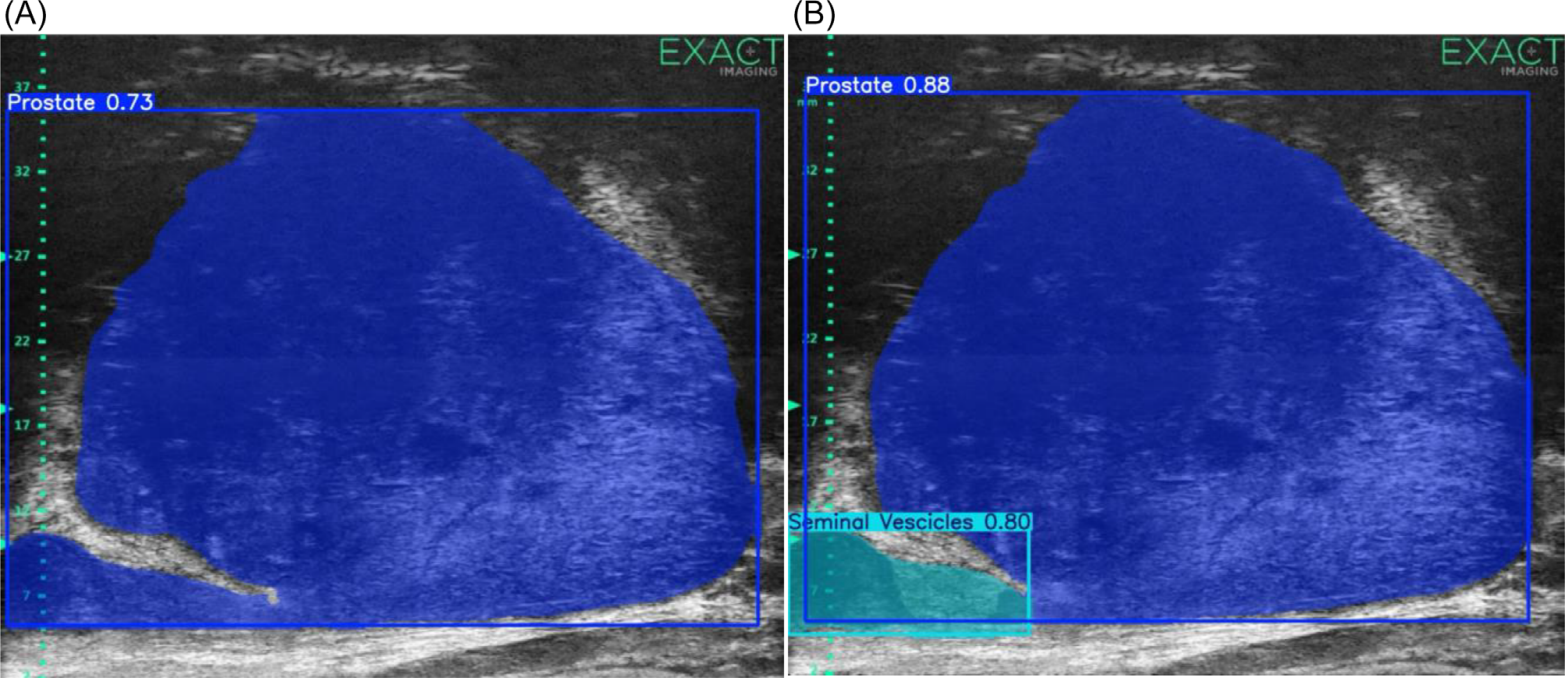
Qualitative comparison of segmentation overlays on unseen test frames. (A) Output from the standard training regime showing sharper but unstable contours. (B) Output from the augmentation‐rich model, demonstrating smoother, temporally consistent and anatomically coherent segmentation.

To complement quantitative metrics, both MUSeg‐PSV models were evaluated on unseen full‐length MicroUS videos from the held‐out test partition, which were never used during training or for early stopping decisions. Figure 4 presents representative test frames with segmentation overlays at 40% opacity (prostate = blue, seminal vesicles = light blue).

The standard model produced sharp but temporally unstable prostate contours and inconsistent seminal vesicle detection. In contrast, upon qualitative inspection, the augmentation‐rich MUSeg‐PSV exhibited smoother, temporally coherent contours and more reliable seminal vesicle delineation, even under low‐contrast or heterogeneous imaging conditions.

## 4 DISCUSSION

Our study demonstrates the technical feasibility of real‐time, dual‐class segmentation of high‐resolution MicroUS images, providing consistent delineation of both the prostate gland and the seminal vesicles at clinically viable frame rates. Taken together, these results position MUSeg‐PSV as a technical feasibility demonstration of anatomically comprehensive MicroUS segmentation, achieved under realistic acquisition and inference constraints, offering a foundation for broader clinical integration and future validation studies.

To ensure robustness under variable imaging conditions, two training regimes were explored. The augmentation‐rich configuration yielded more consistent performance and better generalisation, indicating that introducing spatial and photometric variability during training can improve model stability across heterogeneous acquisition conditions without compromising real‐time performance. While the augmented model showed a reduction in prostate AP (0.967 → 0.548), this drop is attributable to a small number of low‐confidence false‐positive detections that disproportionately penalise the area under the precision–recall curve. In a clinical decision‐making context, such low‐confidence predictions would typically be filtered by standard confidence thresholds (≈ 0.45–0.65), which in our analysis restored precision–recall balance without sacrificing overlap accuracy. Crucially, the prostate DSC improved from 0.675 to 0.770, indicating that the model's actual spatial accuracy on true detections was enhanced, not degraded. Therefore, the AP decrease reflects a statistical artefact of the evaluation metric rather than a clinically meaningful loss of segmentation quality.

Beyond accuracy, model reliability and calibration are essential for safe clinical adoption. MUSeg‐PSV achieved well‐aligned confidence estimates, with the ECE decreasing from 0.56 to 0.16 and the Brier score for the seminal vesicles improving from 1.00 to 0.20. Such calibration ensures that when the model reports high confidence, the segmentation is likely accurate, an important safeguard for intraprocedural applications, where overconfident predictions could mislead interpretation. The marginal decrease in seminal vesicle DSC (0.892 → 0.876) reflects the trade‐off inherent in augmentation‐driven recall improvement: The standard model produced fewer but higher overlap SV predictions, whereas the augmented model detected seminal vesicles more consistently across frames, resulting in a slight dilution of per‐prediction DSC but a substantially higher overall detection rate (AP: 0.183 → 0.842). This trade‐off is clinically favourable, as consistent detection of seminal vesicles across frames is more relevant to intraprocedural guidance than marginal gains in per‐mask overlap. Qualitative assessments further confirmed temporally smooth contours and stable delineation even in low‐contrast regions, suggesting that the model maintains robustness under realistic imaging conditions.

From an engineering perspective, the model's real‐time inference (< 3.5 ms/frame) and low hardware requirements (NVIDIA T4/A100) make it potentially compatible with existing MicroUS consoles. The smooth temporal behaviour may also reduce visual fatigue and facilitate continuous interpretation during scanning, features that are desirable for integration into high‐throughput clinical workflows.

Recent comparative studies suggest that AI‐enhanced MicroUS systems can match or surpass mpMRI in diagnostic performance and workflow efficiency.^1^, ^2^, ^5^ Within this evolving landscape, our findings support the feasibility of using lightweight, real‐time segmentation networks to enhance anatomical feedback during MicroUS examinations, without imposing additional computational burden.

Prior work on MicroUS segmentation remains limited and largely offline. Van den Kroonenberg et al.^15^ achieved high DSC (~0.94) for zonal delineation on 3D CEUS/MicroUS volumes, but inference required ~7.6 s per volume, precluding real‐time use. More recently, Jiang et al. (MicroSegNet)^14^ reported whole‐prostate segmentation with DSC 0.94 and HD95 2.0 mm, though their approach lacked seminal vesicle or zonal annotations and was not designed for procedural deployment.

Against this background, MUSeg‐PSV achieves prostate DSC 0.77 and seminal vesicle AP 0.84 at 35 fps, representing, to our knowledge, the first MicroUS segmentation framework to combine anatomical breadth with real‐time performance at clinically viable frame rates.

These results indicate that MUSeg‐PSV provides a technically sound framework for anatomically guided MicroUS interpretation, bridging the gap between algorithmic development and procedural integration. Although lesion‐level analysis and local‐stage quantification were beyond the scope of this work, the ability to delineate seminal vesicles reliably in real time offers a foundation for future developments. According to the TNM classification, invasion of the seminal vesicles defines stage T3b disease, a distinction that directly impacts clinical management. Its real‐time delineation on MicroUS can influence the surgical strategy for radical prostatectomy, particularly regarding the extent of nerve‐sparing, guiding the decision between intrafascial, interfascial and extrafascial dissection planes. In addition, reliable SV visualisation could prompt targeted biopsies at the prostate base and seminal vesicle junction, improving pre‐operative staging accuracy. Although MUSeg‐PSV currently operates on 2D sagittal frames, the sequential nature of MicroUS sweep acquisitions lends itself to volumetric reconstruction. Stacking frame‐level segmentations along the sweep trajectory could enable 3D visualisation of the prostate and seminal vesicles, supporting multiplanar assessment and spatial mapping of tumour location. Similarly, the framework could be extended to assess extracapsular extension and neurovascular bundle involvement. These directions will be explored in future work. By providing stable, calibrated overlays, the system may support operator orientation and contribute to reducing interobserver variability in MicroUS interpretation.

Real‐time anatomical segmentation on MicroUS has tangible clinical implications. By delivering stable, continuously updated overlays of the prostate and seminal vesicles, MUSeg‐PSV has the potential to assist urologists in maintaining anatomical orientation and targeting accuracy during biopsy, without interrupting workflow. The model's calibrated confidence output could further support less‐experienced operators by highlighting uncertain regions, promoting safer and more consistent guidance. In practice, these features may facilitate more standardised sampling and improved reproducibility across institutions, which are essential for the broader adoption of MicroUS‐guided interventions.

From a deployment standpoint, MUSeg‐PSV requires minimal additional infrastructure: The inference pipeline runs in real time on a standard workstation equipped with a widely available GPU, with no hardware modifications to the existing ExactVu console. Given the model's lightweight architecture and standard dependencies, clinical deployment could be realistically achieved within a limited development cycle, with marginal incremental cost over the existing MicroUS setup.

### 4.1 Limitations

This study has several limitations. First, the dataset was relatively small and derived from a single institution using a single‐vendor transducer, which may limit generalizability and warrants validation on multicenter, multivendor datasets. Second, the seminal vesicle class was underrepresented, potentially affecting segmentation consistency in anatomically complex regions and biassing performance estimates in this substructure. Third, the scope is restricted to anatomical segmentation; lesion‐level outputs and formal staging analyses (including T3b) were not pursued and will require dedicated labels and multimodal ground truth to assess the impact of MUSeg‐PSV on clinically relevant endpoints. Finally, although MUSeg‐PSV achieved stable frame‐level segmentation, temporal smoothness was not quantified with sequence‐level metrics, and future work should incorporate dedicated temporal consistency measures and user‐in‐the‐loop evaluations to better characterise real‐time behaviour in procedural settings.

## 5 CONCLUSION

This study introduces MUSeg‐PSV, to our knowledge, the first real‐time deep‐learning framework for simultaneous segmentation of the prostate gland and seminal vesicles on high‐frequency MicroUS. The model achieved clinically meaningful segmentation performance, robust calibration and real‐time throughput compatible with intraprocedural biopsy guidance. By enabling consistent, on‐screen delineation of seminal vesicles, MUSeg‐PSV lays the groundwork for future automated assessment of extracapsular extension, an essential element of PCa staging and risk stratification. Although multiparametric MRI remains the reference standard, these results suggest that MicroUS augmented with real‐time AI‐based anatomical guidance could represent a viable, accessible adjunct for precision‐guided intervention and diagnostic support in PCa care, with the potential to reduce the learning curve for MicroUS interpretation and support more consistent decision‐making, pending prospective validation.

## AUTHOR CONTRIBUTIONS

*Conceptualization*: Giacomo Cavadini. *Methodology*: Giacomo Cavadini, Luca Di Stefano, Marco Paciotti and Ludovica Cella. *Formal analysis*: Giacomo Cavadini, Luca Di Stefano and Ludovica Cella. *Data curation*: Pier Paolo Avolio, Vittorio Fasulo, Andrea Piccolini, Cesare Saitta and Edoardo Beatrici. *Writing—original draft preparation*: Ludovica Cella. *Writing—review and editing*: Marco Paciotti, Rafal Kocielnik, Andrew J. Hung, Alberto Saita, Paolo Casale and Massimo Lazzeri. *Supervision*: Giovanni Lughezzani and Nicolò Maria Buffi. All authors have read and agreed to the published version of the manuscript.

## CONFLICT OF INTEREST STATEMENT

The authors have nothing to disclose.

## Supporting information


**Table S1.** Patients' baseline characteristics.

## Data Availability

Giovanni Lughezzani has full access to all the data in the study and takes responsibility for the integrity of the data and the accuracy of the data analysis.
