# Supplementary material for: MUSeg‐PSV: A Real‐Time Deep‐Learning Segmentation of the Prostate Gland and Seminal Vesicles on 29‐MHz Micro‐Ultrasound
Source: BJUI Compass. 2026 May 20;7(5):e70211. doi: 10.1002/bco2.70211 (PMC13239491; doi:10.1002/bco2.70211)
Supplement: Supplementary file 1 — Table S1. Patients' baseline characteristics. [file BCO2-7-e70211-s001.docx]

This supplementary material has been provided by the authors to give readers additional

information about their work.

**eTable 1.** Patients’ baseline characteristics

| **Patient** | **DRE** | **Prostate Volume (ml)** | **Medium Lobe** | **PSA (ng/ml)** | **PRIMUS MicroUS** | **ISUP Biopsy** | **PIRADS mpMRI** |
| --- | --- | --- | --- | --- | --- | --- | --- |
| 1 | Negative | 30 | Yes | 5.2 | 2 | - | - |
| 2 | Negative | 65 | No | 4.7 | 2 | - | - |
| 3 | Negative | 80 | No | 7.3 | 2 | - | - |
| 4 | Negative | 40 | Yes | 4.9 | 2 | - | - |
| 5 | Positive | 50 | No | 8.5 | 4 | 2 | 3 |
| 6 | Negative | 70 | No | 5.8 | 3 | - | - |
| 7 | Positive | 85 | Yes | 10.2 | 5 | 4 | 4 |
| 8 | Negative | 35 | No | 4.0 | 2 | - | - |
| 9 | Positive | 50 | No | 9.3 | 3 | 3 | 4 |
| 10 | Negative | 65 | No | 5.5 | 3 | - | 3 |
| 11 | Negative | 40 | Yes | 4.2 | 2 | - | - |
| 12 | Negative | 35 | No | 4.0 | 2 | - | - |
| 13 | Positive | 40 | No | 7.8 | 4 | 2 | 3 |
| 14 | Negative | 30 | Yes | 5.1 | 3 | - | 3 |

*DRE=Digital Rectal Examination, PSA=Prostate-Specific Antigen, PRIMUS=Prostate Risk Identification using Micro-Ultrasound, ISUP=*International Society of Urological Pathology *Grade Group, PIRADS=Prostate Imaging Reporting and Data System, mpMRI=Multiparametric MRI, microUS=Micro-ultrasound*
